# Investigating mechanical properties and biocement application of CaCO_3_ precipitated by a newly-isolated *Lysinibacillus* sp. WH using artificial neural networks

**DOI:** 10.1038/s41598-020-73217-7

**Published:** 2020-09-30

**Authors:** Jindarat Ekprasert, Ittipon Fongkaew, Poemwai Chainakun, Rungtiwa Kamngam, Wachiraya Boonsuan

**Affiliations:** 1 Department of Microbiology, Faculty of Science, Khon Kaen University, 123 Mitraparp Road, Muang, Khon Kaen, 40002 Thailand; 2 School of Physics, Institute of Science, Suranaree University of Technology, Nakhon Ratchasima, 30000 Thailand

**Keywords:** Biotechnology, Microbiology, Engineering, Materials science, Physics

## Abstract

A newly-isolated *Lysinibacillus* sp. strain WH could precipitate CaCO_3_ using calcium acetate (Ca(C_2_H_3_O_2_)_2_), calcium chloride (CaCl_2_) and calcium nitrate (Ca(NO_3_)_2_) via non-ureolytic processes. We developed an algorithm to determine CaCO_3_ crystal structures by fitting the simulated XRD spectra to the experimental data using the artificial neural networks (ANNs). The biogenic CaCO_3_ crystals when using CaCl_2_ and Ca(NO_3_)_2_ are trigonal calcites with space group R3c, while those when using Ca(C_2_H_3_O_2_)_2_ are hexagonal vaterites with space group P6_5_22. Their elastic properties are derived from the Voigt–Reuss–Hill (VRH) approximation. The bulk, Young's, and shear moduli of biogenic calcite are 77.812, 88.197, and 33.645 GPa, respectively, while those of vaterite are 67.082, 68.644, 25.818 GPa, respectively. Their Poisson’s ratios are ~ 0.3–0.33, suggesting the ductility behavior of our crystals. These elastic values are comparable to those found in limestone cement, but are significantly larger than those of Portland cement. Based on the biocement experiment, the maximum increase in the compressive strength of Portland cement (27.4%) was found when Ca(NO_3_)_2_ was used. An increased strength of 26.1% was also found when Ca(C_2_H_3_O_2_)_2_ was used, implying the transformation of less-durable vaterite to higher-durable calcite. CaCO_3_ produced by strain WH has a potential to strengthen Portland cement-based materials.

## Introduction

Cement is one of the most used construction materials due to its durability and being widely affordable. An increase in cement consumption has led to a negative impact on the global environment. The CO_2_ emission from the production of cement-based materials in industries has constituted up to 3.4% of the total CO_2_ global emission and 8–10% of global anthropogenic CO_2_ emissions^1^. The inevitable consequences of long-term usage of the cement-based materials are the formation of microcracks within the infrastructure due to exposure to environmental factors such as temperature changes, corrosive substances, and external loads. These factors then affect mechanical properties of the materials including compressive strength, tensile strength and permeability^2^. Traditional approaches which have been used to repair cracked materials are, for example, the use of epoxy resin or polyurethane to seal the cracks. This way may lead to secondary cracking because those chemicals have different coefficients of thermal expansion to cement-based materials^3^. Therefore, alternative repair technology is needed to widely investigate in order to reduce environmental impacts.

Recently, many research has focused on an application of biological substances to enhance mechanical properties of cement, specifically strength improvement via bacterial mineralization called microbially induced calcium carbonate precipitation (MICP)^4^. Under suitable conditions bacteria are capable of influencing calcium carbonate (CaCO_3_) precipitation during their metabolic processes. Benefits of using calcifying microorganisms to improve mechanical properties of cement are that these microbes can heal the cracks from the inside, resulting in homogeneity of the repaired materials. Moreover, biological approach is often more environmentally-friendly since no toxic chemicals or high consumption of energy is required like what is needed for the chemical and physical approaches, respectively^5,6^.

There are two types of bacterial metabolisms involved in MICP, urea hydrolysis and non-ureolytic processes such as ammonification of amino acids, denitrification, dissimilatory of sulfate reduction and photosynthesis^7,8^. The release of metabolites from these reactions can create a high pH environment, which is favorable to CaCO_3_ precipitation. Bacterial cells then act as nucleation sites for CaCO_3_ crystal formation^9^. Among these metabolic processes, urea hydrolysis is less complex and thus its pathway is the most widely studied^10^. Nevertheless, urea hydrolysis produces ammonia as a byproduct causing an increased risk of steel corrosion in reinforced concrete^11^. Therefore, non-ureolytic reactions become more interesting for actual applications. In this work, we then focus on the growth and potential application of calcifying bacteria which can produce CaCO_3_ in the absence of urea.

CaCO_3_ precipitated by bacteria can be used for the biodeposition treatment of engineering constructions. Chahal and Siddique^12^ found that *Sporosarcina pasteurii* in the concrete could seal the cracked surface through calcite production. However, the long-term performance of the repaired materials depends on the crystal structure of the deposited CaCO_3_. There are several factors influencing the formation of CaCO_3_ by bacteria including calcium sources, pH, dissolved inorganic carbon and availability of nucleation sites^13,14^. De Muynck et al.^15^ discovered that the composition of nutrients during biodeposition of CaCO_3_ within the cement and bacterial strains has significantly influenced the morphology of CaCO_3_ crystals^16^. The crystal morphology could be, for example, rhombohedral, orthorhombic, hexagonal and spheroid^17^. The potential use of calcium chloride (CaCl_2_), calcium acetate (Ca(CH_3_COO)_2_) and calcium nitrate (Ca(NO_3_)_2_) for improving the durability and repairing cracks of concrete was also reported^18^. Many studies showed the benefit of MICP to strengthen cement and concrete by applying CaCl_2_ as a calcium source. However, it is commonly known that chloride penetration will cause the depassivation and electrochemical corrosion of the embedded steel reinforcement, and thus negatively affect durability of the materials^3^. In this work, we attempted to compare the use of different calcium sources, including Ca(CH_3_COO)_2_, CaCl_2_ and Ca(NO_3_)_2_, for bacterial growth and calcification in order to suggest alternative calcium compound(s) to replace CaCl_2_ for actual engineering applications by our strain.

The scope of this work was to investigate the ability of a newly-isolated *Lysinibacillus* sp. strain WH to precipitate CaCO_3_ using different calcium sources as was previously mentioned. The aim was to characterize the crystal structure of the obtained CaCO_3_. The sizes and structures of CaCO_3_ crystals can greatly affect the physical properties of cementitious composites such as the compressive strength, the durability, and the cement hydration^19^. In this study, we developed an algorithm that employed the artificial neural networks to determine the crystal structures of CaCO_3_. The corresponding elastic parameters including the bulk modulus, Young's modulus, shear modulus and Poisson's ratio were predicted based on Voigt–Reuss–Hill (VRH) approximation^20^. The quality of CaCO_3_ obtained in this work was also experimented as a mixture in Portland cement. The comparison of the compressive strength of Portland cement with and without strain WH inoculation were discussed.

## Methods

### Bacterial isolation and identification

Saline soil samples were collected from an abandoned paddy field in Surin, Thailand (14°54′14.2″N 103°53′29.1″E). Ten grams of samples were mixed with B4 medium (per liter: 4 g yeast extract, 5 g dextrose, 2.5 g calcium acetate and 1.5% (w/v) agar for solid medium, pH 8.2)^21^. Soil suspension was shaken at 150 rpm, 30 °C, 7 days. The serially-diluted suspension was plated onto B4 agar. Colonies developed white deposits were subcultured onto fresh B4 agars until pure cultures were obtained. Stock cultures were kept in 20% (v/v) glycerol at -80 °C for further experiment.

Genomic DNA of the isolate was extracted by using a Bacterial DNA Extraction Kit (Vivantis, Malaysia) following manufacturer’s instructions. PCR amplification of 16S rRNA genes was carried out using a universal PCR primer pairs for the bacterial 16S rRNA gene as follows: 27F (5′-AGAGTTTGATCCTGGCTCAG-3′) and 1492R (5′-GGTTACCTTGTTACGACTT-3′)^22^. The reaction mixture consisted of 50 ng of template DNA, 1 × Taq polymerase buffer, 20 pmol of each primer, 2.5 mM of dNTPs, 2.5 U of Taq DNA polymerase and DNase-free water was added to a total volume of 50 µl. PCR conditions were 94 °C for 5 min, and then 94 °C for 1 min, 56 °C for 1 min, 72 °C for 1 min, followed by 72 °C for 10 min, 30 cycles. PCR products were verified by a gel electrophoresis and were then purified using an AmbiClean kit (Vivantis, Malaysia) following manufacturer’s instructions. DNA was quantified using a micro-volume spectrophotometer (Maestrogen, Taiwan). Purified PCR products were submitted to Macrogen (Republic of Korea) for sequencing. The 16S rRNA gene sequences were identified by BLAST against the sequences on the Genbank database on NCBI. Phylogenetic relationships of the 16S rRNA gene sequences were constructed using MEGA 6.0 software^23^.

### Growth of a bacterial isolate using different calcium sources

Ca(CH_3_COO)_2_, Ca(NO_3_)_2_ and CaCl_2_ which are common admixtures used in engineering materials were used as sources of calcium ions. B4 medium was modified by replacing Ca(CH_3_COO)_2_ in the medium with other calcium compounds mentioned earlier. The pH of the medium was initially adjusted to 7.0 in all experiments, which were performed in triplicate each. Non-inoculated flasks were set up as a control. Cultures were incubated at 30 °C, 150 rpm for 7 days. Samples were collected every 12–24 h for bacterial growth measurement by plate count method.

### Quantification of the precipitated CaCO_3_

Precipitates formed in bacterial cultures and in abiotic flasks were collected after 7 days of incubation. Precipitates were harvested by filtering through a pre-weighed filter paper (Whatman no.1, Merck, Germany). Solid samples were washed twice with sterile water to remove unattached cells from the precipitates. The samples were then dried in a hot-air oven at 45 °C until completely dry. Weights of precipitates were calculated as follows:

1 $$ W_{precipitates} = W_{Total} - W_{paper} , $$

where *W*_*precipitates*_, *W*_*Total*_ and *W*_*paper*_ are the weights of precipitates collected from inoculated flask, of filter paper containing precipitates, and of empty filter paper, respectively. After quantification, precipitates were kept in a desiccator for further analysis by SEM and XRD. For comparison, biogenic CaCO_3_ after 7 days of incubation were collected by centrifugation at 8000 rpm, 10 min, 4 °C. The precipitates were freeze-dried using a lyophilizer (Labconco, USA) at − 80 °C. The resulting dehydrated bacterial CaCO_3_ were subjected to SEM observation.

### Scanning electron microscope (SEM) analysis

Morphology of precipitates were observed using a Leo 1450VP scanning electron microscope (SEM) (Zeiss, Germany) at an accelerating voltage of 15–30 kV. Samples were sputter coating with gold prior to examination.

### X-ray powder diffraction (XRD) analysis

Mineralogy of the precipitates were analyzed by using powder X-ray diffractometer (Malvern Panalytical Empyrean, UK) with CuK radiation source at 30 kV/30 mA. Spectra were scanned from 20°–60°. XRD diffractograms were analyzed using an algorithm developed through the use of artificial neural networks.

### Analyzing crystal structures using artificial neural networks (ANNs)

To determine the possible crystal structures of biogenic CaCO_3_, we considered all types of the CaCO_3_ crystal structure available in the Materials Project Database^24^, as summarized in Table 1. Different crystal structures associated with different space groups produce different characteristic signatures on the XRD profiles. CaCO_3_ in each space group also requires different formation energy to form its crystal structure. The lower the formation energy is, the higher chance the crystal forms in that space group.

**Table 1:** Details of each type of CaCO_3_ crystal structure available in the Materials Project Database^24^.

Materials Id	Space group	Formation energy (eV)	Band gap (eV)	Volume	N sites	Density (g/cc)
mp-3953	R3c	− 2.707	5.002	127.17	10	2.614
mp-556235	P2_1_/c	− 2.705	4.960	253.066	20	2.627
mp-561412	C2	− 2.7	4.958	191.628	15	2.602
mp-560265	P6_5_22	− 2.699	4.944	1174.591	90	2.547
mp-1194399	C2/c	− 2.699	4.910	389.315	30	2.561
mp-1197939	P1	− 2.698	4.943	1170.318	90	2.556
mp-553939	Pnma	− 2.694	4.700	258.226	20	2.574
mp-1079918	P1	− 2.691	4.849	124.855	10	2.662
mp-4626	Pnma	− 2.683	4.171	234.244	20	2.838
mp-1197230	P1	− 2.683	4.940	591.983	50	2.807
mp-3205	Pmmn	− 2.537	4.257	110.731	10	3.002
mp-548403	C222_1_	− 2.395	5.719	105.128	10	3.162
mp-641635	Pnma	− 1.338	1.185	306.458	20	2.169
mp-696740	C2/c	− 0.689	1.435	118.88	10	2.796

Their materials Id, space group as well as corresponding formation energy are presented in the first, second, and third columns, respectively. The corresponding values of band gap and volume of unit cells are presented in the fourth and fifth columns, respectively. The values of N sites reported in the sixth column represent the number of atoms per unit cell. The density of each crystal structure is shown in the last column.

**Figure 1:**
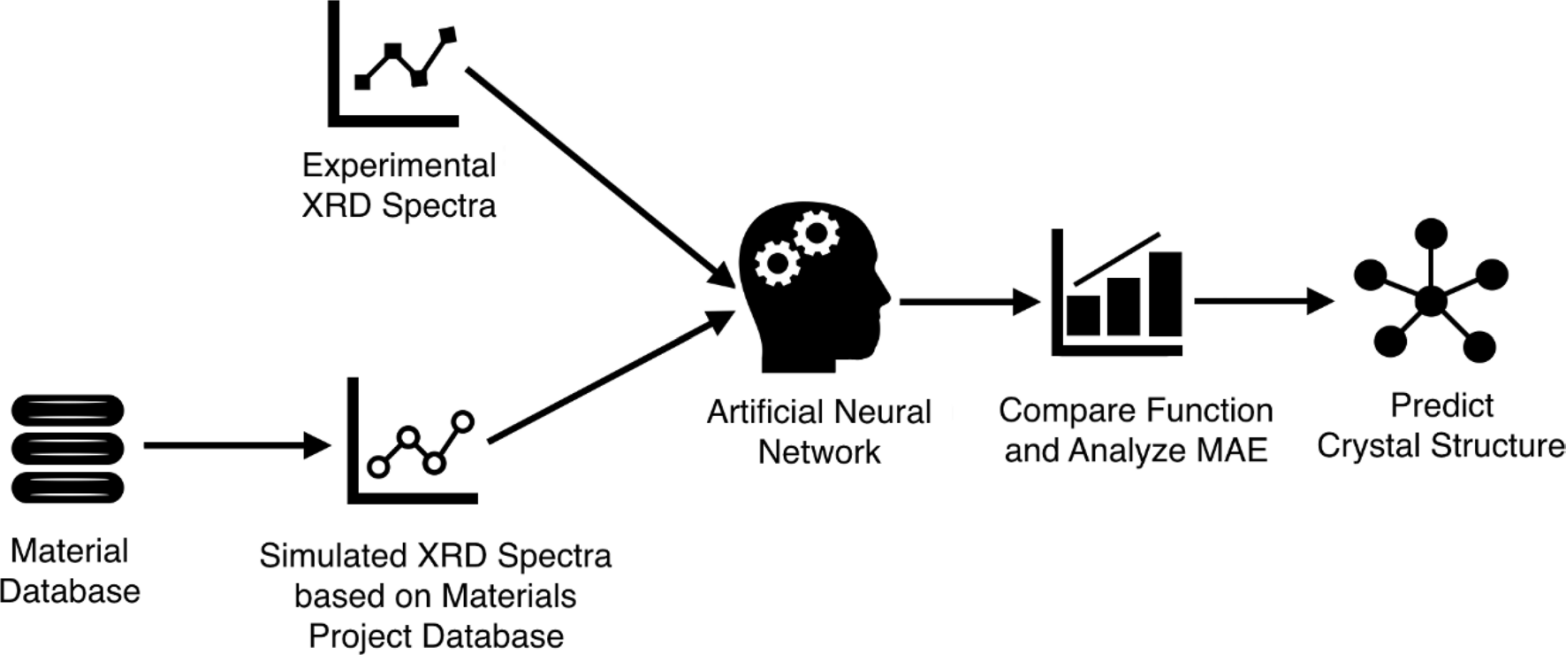
Process for matching simulated XRD patterns with experimental XRD patterns in order to determine the crystal structures of CaCO_3_.

We designed an algorithm that could sort the formation energy from the database and simulate the corresponding XRD spectra from the lattice, and atomic structure parameters that are implemented in VESTA utilizes RIETAN-FP^25,26^. The simulated XRD spectra are fitted to our experimental XRD data and are weighted by formation energy. The fitting was performed using an artificial neural network method by building mathematical function of normalized intensity and 2θ in order to compare the mean absolute error (MAE) per formation probability. For each biogenic CaCO_3_, its predicted crystal structure was the one that provides the lowest MAE per formation probability. The process for determining the crystal structures of biogenic CaCO_3_ was summarized in Fig. 1.

### Calculating elastic parameters of biogenic CaCO_3_

The elastic properties of our biogenic CaCO_3_ were derived from the Voigt–Reuss–Hill (VRH) approximation^20^, a useful scheme to convert elastic constants of the single crystals into macroscopic elastic moduli for the corresponding bulk materials. We focused on key elastic properties inferred by the bulk, Young's, and shear moduli, and the Poisson's ratio. The bulk modulus (*B*) is a measure of the incompressibility of material under pressure on all surfaces while the Young modulus (*E*) is a parameter that measures the stiffness of a material. The shear modulus (*G*) is the modulus of rigidity explaining the deformation of the material due to shearing or torsion force. The Poisson's ratio (*ν*) is a measure of the Poisson effect in which a shape of material tends to change in directions perpendicular to the direction of an applied force.

The single-crystal elastic constants could be determined by using the stress–strain approach from first-principles calculations performed using the Vienna *Ab* initio Simulation Package (VASP)^25^. There are five independent elastic constants for the hexagonal structures and seven independent elastic constants for the trigonal structures, denoted by *C*_*11*_, *C*_*12*_, *C*_*13*_, *C*_*14*,_ *C*_*15*_, *C*_*33*_, and *C*_*44*_. The *C*_*44*_ is equivalent to C_55_. The constant *C*_*66*_ is defined as *C*_*66*_ = (*C*_*11*_- *C*_*12*_)/2. The stress–strain relation for the hexagonal and trigonal structures can be written in the matrix form^26^ as,2

For hexagonal and trigonal structures, Voigt–Reuss–Hill (VRH) approximation predicted the polycrystalline bulk modulus (*B*) and shear modulus (*G*) using the calculated elastic constants of the single crystal following the Voigt (*B*_*V*_ and *G*_*V*_) and the Reuss (*B*_*R*_ and *G*_*R*_) approximations^27^. The polycrystalline *B*_*V*_, *G*_*V*_, *B*_*R*_ and *G*_*R*_ approximations can be listed below.

3 $$ B_{V} = 1/9\;\;[2(C_{11} + C_{12} ) + C_{33} + C_{13} ], $$

4 $$ G_{V} = 1/30\;\;[7C_{11} - 5C_{12} + 12C_{44} + 2C_{33} - 4C_{13} ], $$

5 $$ B_{R} = \, [(C_{11} + C_{12} )C_{33} - 2C^{2}_{13} \left] { \, / \, } \right[C_{11} + C_{12} + C_{33} - C_{13} ], $$

6 $$ G_{R} = \{ 5[(C_{11} + C_{12} )C_{33} - 2C^{2}_{13} ]C_{44} C_{66} \} /\{ 6B_{V} C_{44} C_{66} + 2[(C_{11} + C_{12} )C_{33} - 2C^{2}_{13} ](C_{44} + C_{66} )]. $$

These approximations from Voigt^27^ and Reuss^28^ are upper and lower bounds of elastic constants, respectively. Therefore, Hill’s averages are used to predict bulk and shear moduli of the polycrystalline aggregates by

7 $$ B_{H} = 1/2(B_{R} + B_{V} ) \quad {\text{and}}\quad G_{H} = 1/2(G_{R} + G_{V} ). $$

The Young’s modulus and Poisson’s ratio are given by the following formulas:

8 $$ E = 9BG/(3B + G) \quad {\text{and}}\quad { }\upsilon = (3B - 2G)/(2(3B + G)). $$

### Preparation of biocement and compressive strength test

*Lysinibacillus* sp. strain WH was grown with different calcium sources to reach its exponential phase. An initial cell concentration of 1 × 10^10^ CFU/ml was added to each cement cube. Cement was mixed with water at a water:cement ratio of 0.5. Cement paste was casted into a 70 × 70 × 70 mm mold and then was left to harden at room temperature for 24 h. Cement cubes without bacterial inoculation were set up as a control. Each set of cement cube was carried out in triplicate. After demolding, cement cubes were cured in water at ambient temperature for 28 days. Compressive strength of cement cubes was tested according to the ASTM standard^29^ at 0, 7, 14 and 28 days of curing using a CBN Compression testing machine (CBN Testing Corporation, Thailand). Furthermore, biocement fractures were powdered prior to SEM and XRD analysis. A field emission scanning electron microscope (FE-SEM, JEOL JSM 6500F) was used to visualize the morphology of biocement powders. The Reference Intensity Ratio (RIR) analysis^30^ was performed using HighScore Plus Software.

### Statistical analysis

A significant difference among the mean values was analyzed by using Analysis of Variance (ANOVA) and F-test based on the Least Significant Difference test (LSD) at *p*-value < 0.05. All statistical analysis was performed using the Statistix 8.0 program.

## Results

### Isolation and identification

**Figure 2:**
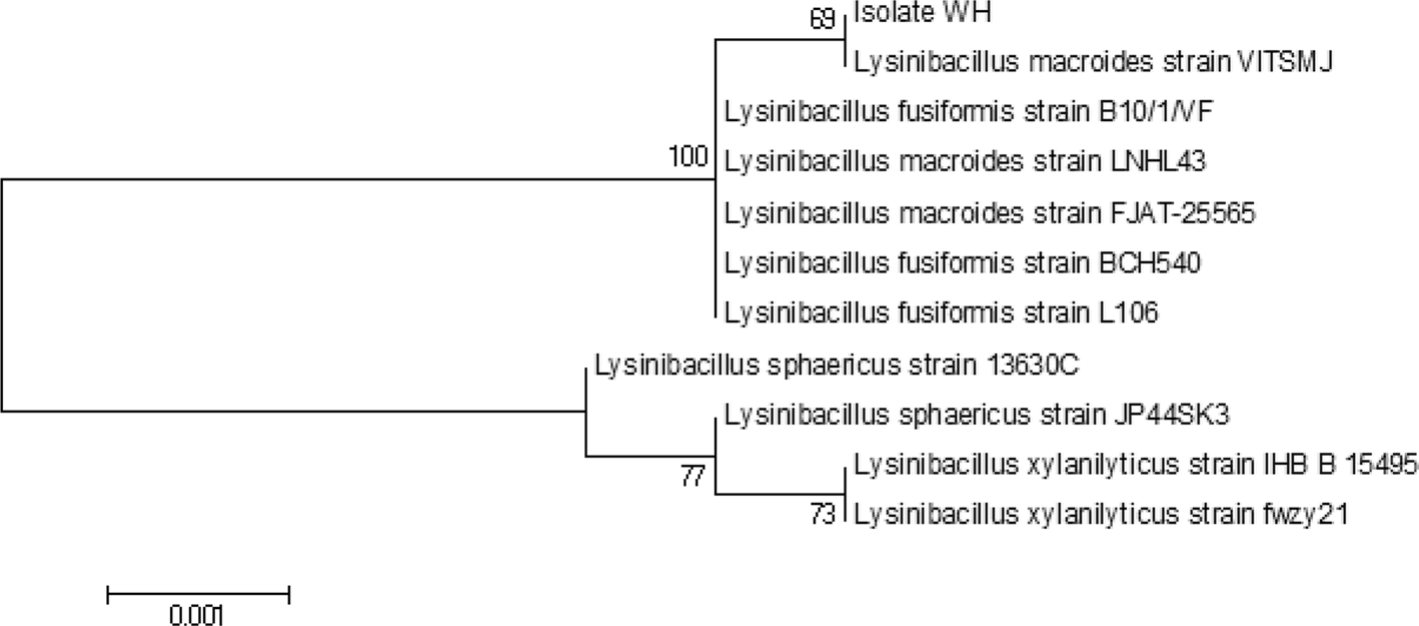
Phylogenetic relationship among the 16S rRNA gene sequence (1420 bp) of isolate WH and the sequences of its closest relatives, constructed using MEGA6 software^23^. Sequence analysis was carried out using a neighbour-joining method with 1000 bootstraps.

A total of two isolates with different morphologies were obtained. Isolate WH was selected based on its ability to provide a higher amount of biomass when grown in different calcium compounds. Genus identification of isolate WH was carried out based on the 16S rRNA gene sequence. Phylogenetic relationship between the 16S rRNA gene sequence of isolate WH and that of its close relatives is shown in Fig. 2. The result showed that isolate WH belongs to the genus *Lysinibacillus* spp., which is most closely related to *Lysinibacillus macroides* strain VITSMJ with 99% identity. Isolate WH cells were stained Gram-positive, rod-shaped, spore-forming. It grows well at 30 °C under aerobic conditions. Isolate WH is, therefore, named as *Lysinibacillus* sp. strain WH throughout this work.

### Growth of *Lysinibacillus* sp. strain WH in B4 medium containing different calcium sources

**Figure 3:**
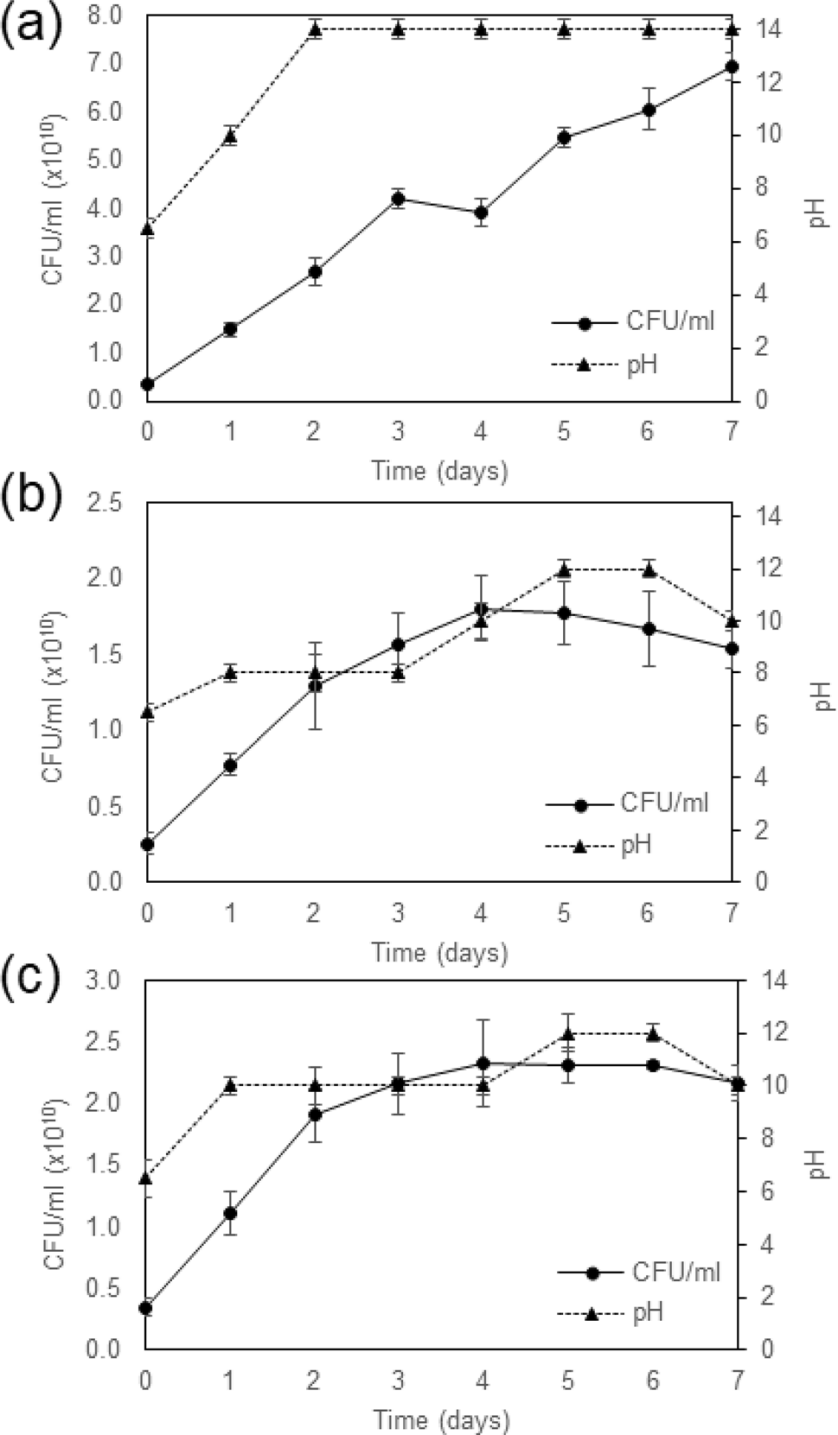
Growth and pH of *Lysinibacillus* sp. strain WH cultures in B4 medium containing different calcium sources—(**a**) calcium acetate, (**b**) calcium chloride and (**c**) calcium nitrate. Error bars represent standard deviations of triplicate data.

To investigate the influence of calcium sources on the growth of *Lysinibacillus* sp. strain WH, cultures were grown in B4 medium containing calcium acetate, calcium chloride or calcium nitrate. According to Fig. 3, the pH increases with increasing viable cell counts in all growth conditions. The highest growth of strain WH (~ 6.5 × 10^10^ CFU/ml) and the highest pH (~ 14) were found when using calcium acetate as a calcium source. The growth of strain WH in calcium chloride and calcium nitrate media was not much different, which was ~ (1.5–2.0) × 10^10^ CFU/ml. The pH was as high as 12 in these two conditions. These results suggested that strain WH was capable of producing an alkaline environment, which was suitable for the CaCO_3_ precipitation.

### Quantification of the precipitated CaCO_3_

**Figure 4:**
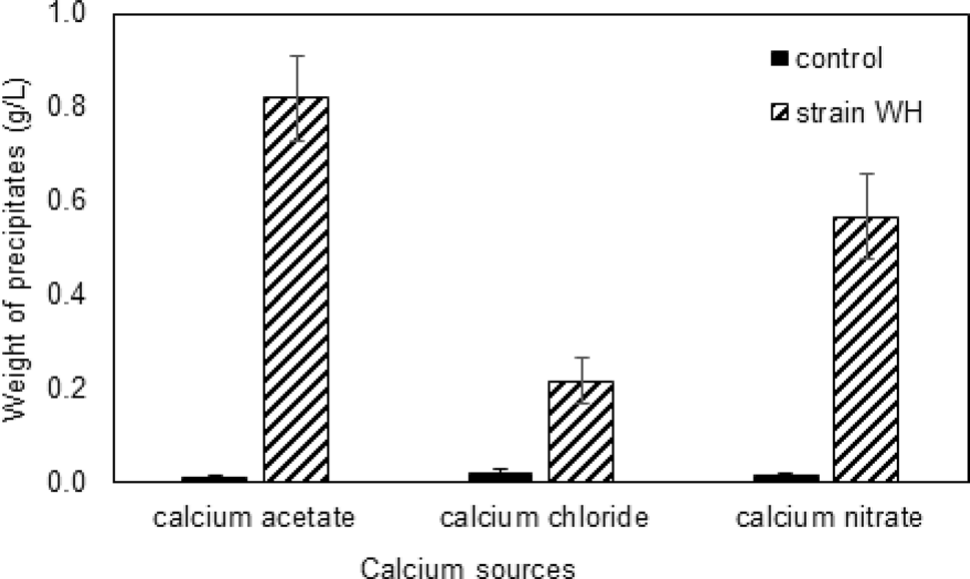
The amount of precipitates collected from *Lysinibacillus* sp. strain WH during growth using different types of calcium sources—calcium acetate, calcium chloride and calcium nitrate. Filled bars represent the precipitates obtained from the non-inoculated controls. Stripped bars represent the precipitates obtained from strain WH cultures. Error bars indicate standard deviations of triplicate data.

The results (Fig. 4) showed that the amount of precipitates in the non-inoculated controls was less than 0.02 g/l, while those in all bacterial cultures were higher than 0.1 g/l. This means that CaCO_3_ precipitation was due to microbial activity, rather than chemical reactions. The CaCO_3_ production was found to be 0.8, 0.2, and 0.6 g/l when strain WH was grown with calcium acetate, calcium chloride, and calcium nitrate, respectively. Therefore, calcium acetate is the best calcium source for the growth of strain WH (Fig. 3) and its CaCO_3_ precipitation (Fig. 4).

### Scanning electron microscopy

**Figure 5:**
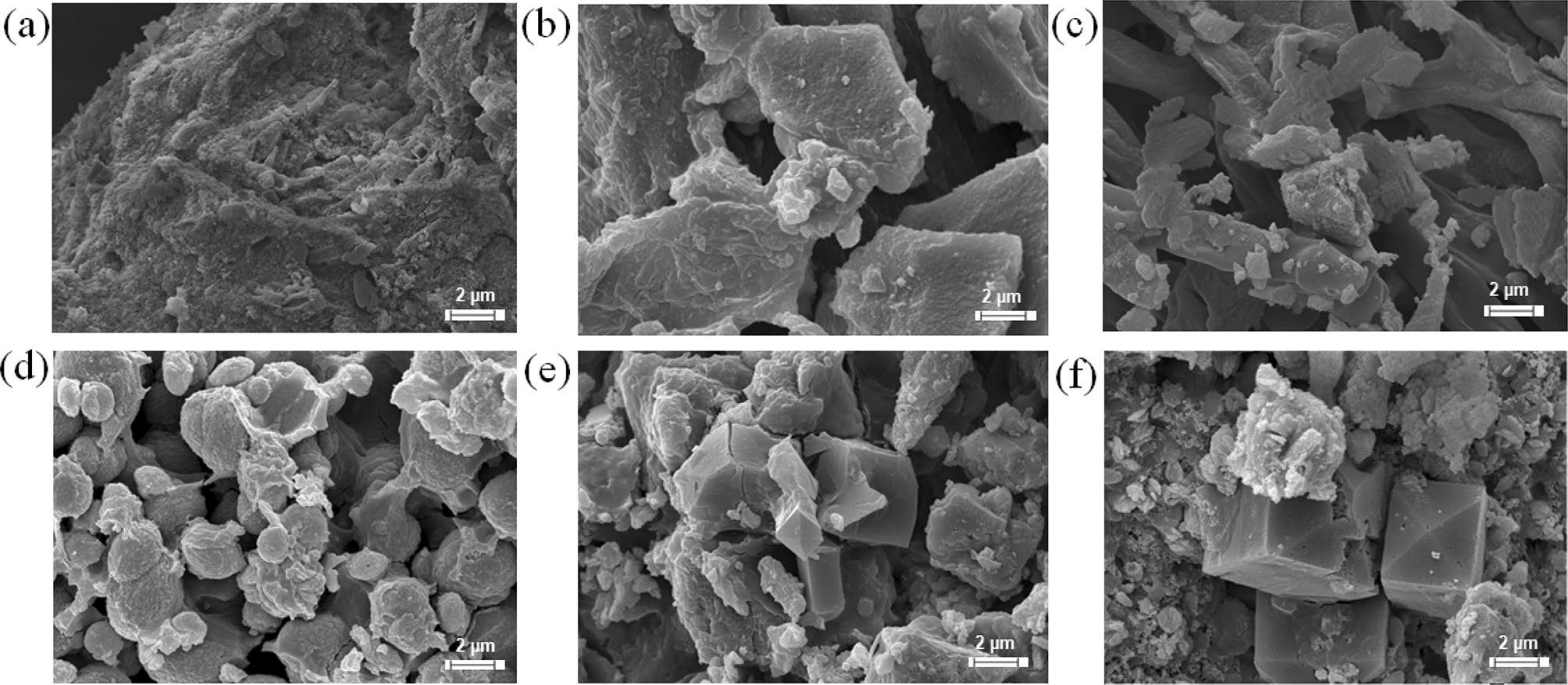
Scanning electron micrographs (× 5000 magnification) of calcium carbonate crystals precipitated by *Lysinibacillus* sp. strain WH grown using different calcium sources—calcium acetate (**d**), calcium chloride (**e**), and calcium nitrate (**f**), compared to precipitates in their corresponding controls showing in (**a**–**c**), respectively. Each picture is a representative of the multiple similar pictures. Scale bars are shown at bottom right.

Precipitates collected from 7-day cultures under different conditions were visualized using SEM. Precipitates collected from abiotic controls (Fig. 5a–c) showed irregular morphology while those obtained from strain WH cultures showed organized crystal morphologies. Rhombohedral crystals were found in strain WH grown using calcium chloride (Fig. 5e) and calcium nitrate (Fig. 5f) as calcium sources. Differently, spheroidal crystals were found when strain WH was grown with calcium acetate (Fig. 5d). Therefore, calcium sources play an important role in shaping crystal morphologies. For comparison, the SEM results of freeze-dried biogenic CaCO_3_ were also provided in Supplementary Figure 1. Although a relatively small amount of crystals is observed, the crystal phase form of CaCO_3_ from each different calcium source is not much different compared to what obtained using the oven-dried method. We, however, selected to use the oven-dried biogenic CaCO_3_ for further analysis because its process is less time consuming and more cost effective in the real application than the freeze-dried process.

### Crystal structures of CaCO_3_ analyzed using artificial neural networks

**Figure 6:**
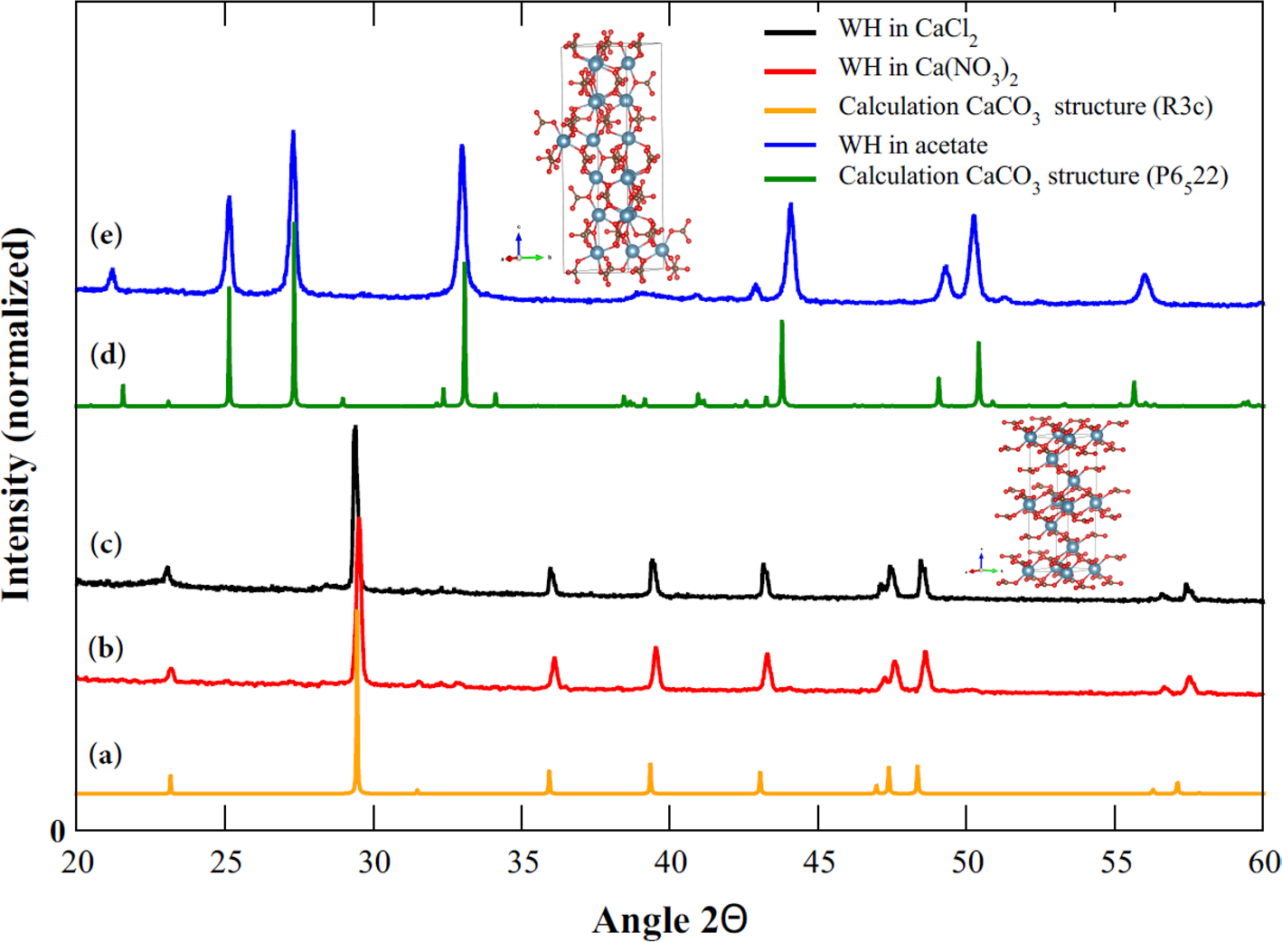
Comparison between the calculated and experimental XRD spectra. (**a**) The calculated XRD spectra of CaCO_3_ structures in trigonal R3c space group or calcite. (**b**,**c**) The experimental XRD spectra of CaCO_3_ obtained from strain WH grown using calcium nitrate and calcium chloride, respectively. (**d**) The calculated XRD spectra of CaCO_3_ structures hexagonal P6_5_22 space group or vaterite. (**e**) The experimental XRD spectra of CaCO_3_ obtained from strain WH grown using calcium acetate.

An algorithm based on artificial neural networks was developed to produce the simulated XRD spectra to fit the experimental data in order to determine the crystal structure of CaCO_3_ precipitated by strain WH (see “Analyzing crystal structures using artificial neural networks (ANNs)” and “Calculating elastic parameters of biogenic CaCO_3_” for the detailed calculations). We found two different spectral models that match with our experimental XRD spectra. This suggested that the obtained CaCO_3_ has two different structures or phases depending on the calcium sources in the bacterial growth medium. The results were shown in Fig. 6. CaCO_3_ crystals precipitated by strain WH in the presence of calcium chloride and calcium nitrate have a trigonal structure belonging to the space group R3c, or calcite, while the CaCO_3_ precipitated by strain WH grown with calcium acetate have a hexagonal structure belonging to the space group P6_5_22, or vaterite. Note that the CaCO_3_ characterized in the space group R3c is commonly found and most stable in nature because it requires lowest formation energy of − 2.707 eV to form (Table 1). Contrarily, the metastable hexagonal phases of CaCO_3_ vaterite is less stable than either calcite or aragonite (orthorhombic phase). Due to its higher solubility, vaterite can convert to calcite at low temperature or aragonite at high temperature of ~ 60 °C^31^. Furthermore, the CaCO_3_ precipitated by strain WH in all growth conditions is also of high purity due to the fact that we did not find other phases mixed in the sample when compared with our structure model.

### Elastic properties of the precipitated CaCO_3_

The elastic properties of each crystal structure calculated using Voigt–Reuss–Hill (VRH) approximation were presented in Tables 2 and 3. Note that the bulk modulus *B* reflects the resistance of materials against volume change. The shear modulus *G* reflects the resistance of materials against shape change. The Young’s modulus *E* measures the stiffness of materials. The crystal structures of CaCO_3_ obtained from strain WH grown with calcium nitrate and calcium chloride are similar, which are in the trigonal space group R3c. We found their bulk modulus, Young's modulus, and shear modulus are 77.812, 88.197, and 33.645 GPa, respectively. On the other hand, CaCO_3_ precipitated by strain WH grown with calcium acetate is in the hexagonal P6_5_22 space group whose bulk modulus, Young's modulus, and shear modulus are found to be 67.082, 68.644, 25.818 GPa, respectively.

**Table 2:** Predicted elastic parameters of CaCO_3_ (space group R3c) obtained from strain WH grown with calcium nitrate and calcium chloride using Voigt–Reuss–Hill (VRH) approximation.

Averaging scheme	Bulk modulus	Young's modulus	Shear modulus	Poisson's ratio
Voigt	*B*_V_ = 81.32 GPa	*E*_V_ = 96.807 GPa	*G*_V_ = 37.188 GPa	*ν*_V_ = 0.302
Reuss	*B*_R_ = 74.309 GPa	*E*_R_ = 79.564 GPa	*G*_R_ = 30.103 GPa	*ν*_R_ = 0.322
Hill	*B*_H_ = 77.814 GPa	*E*_H_ = 88.221 GPa	*G*_H_ = 33.645 GPa	*ν*_H_ = 0.311
Average	77.812	88.197	33.645	0.312

The bulk modulus, Young’s modulus, shear modulus and Poisson’s ratio are listed in the second, third, fourth, and fifth columns, respectively.

**Table 3:** Predicted elastic parameters of CaCO_3_ (space group P6_5_22) obtained from strain WH grown with calcium acetate using Voigt–Reuss–Hill (VRH) approximation.

Averaging scheme	Bulk modulus	Young's modulus	Shear modulus	Poisson's ratio
Voigt	*B*_V_ = 70.051 GPa	*E*_V_ = 70.041 GPa	*G*_V_ = 26.265 GPa	*ν*_V_ = 0.333
Reuss	*B*_R_ = 64.113 GPa	*E*_R_ = 67.243 GPa	*G*_R_ = 25.371 GPa	*ν*_R_ = 0.325
Hill	*B*_H_ = 67.082 GPa	*E*_H_ = 68.647 GPa	*G*_H_ = 25.818 GPa	*ν*_H_ = 0.329
Average	67.082‬	68.644	25.818‬	0.329

The bulk modulus, Young’s modulus, shear modulus and Poisson’s ratio are listed in the second, third, fourth, and fifth columns, respectively.

Moreover, the brittle and ductility nature of the hexagonal and trigonal were analyzed according to the Poisson’s ratio (*v*). The ductility and brittleness of materials are separated by the Poisson's ratio with the critical value of 0.26. If the Poisson's ratio is less than 0.26, the materials are predicted to have brittle behavior; otherwise, materials should behave in a ductile manner^32^. From Tables 2 and 3, our results showed that the Poisson’s ratio is around 0.3–0.33 for both cases which are greater than 0.26. These values indicated that our crystal showed behavior as a ductile material.

### Compressive strength of biocement

**Figure 7:**
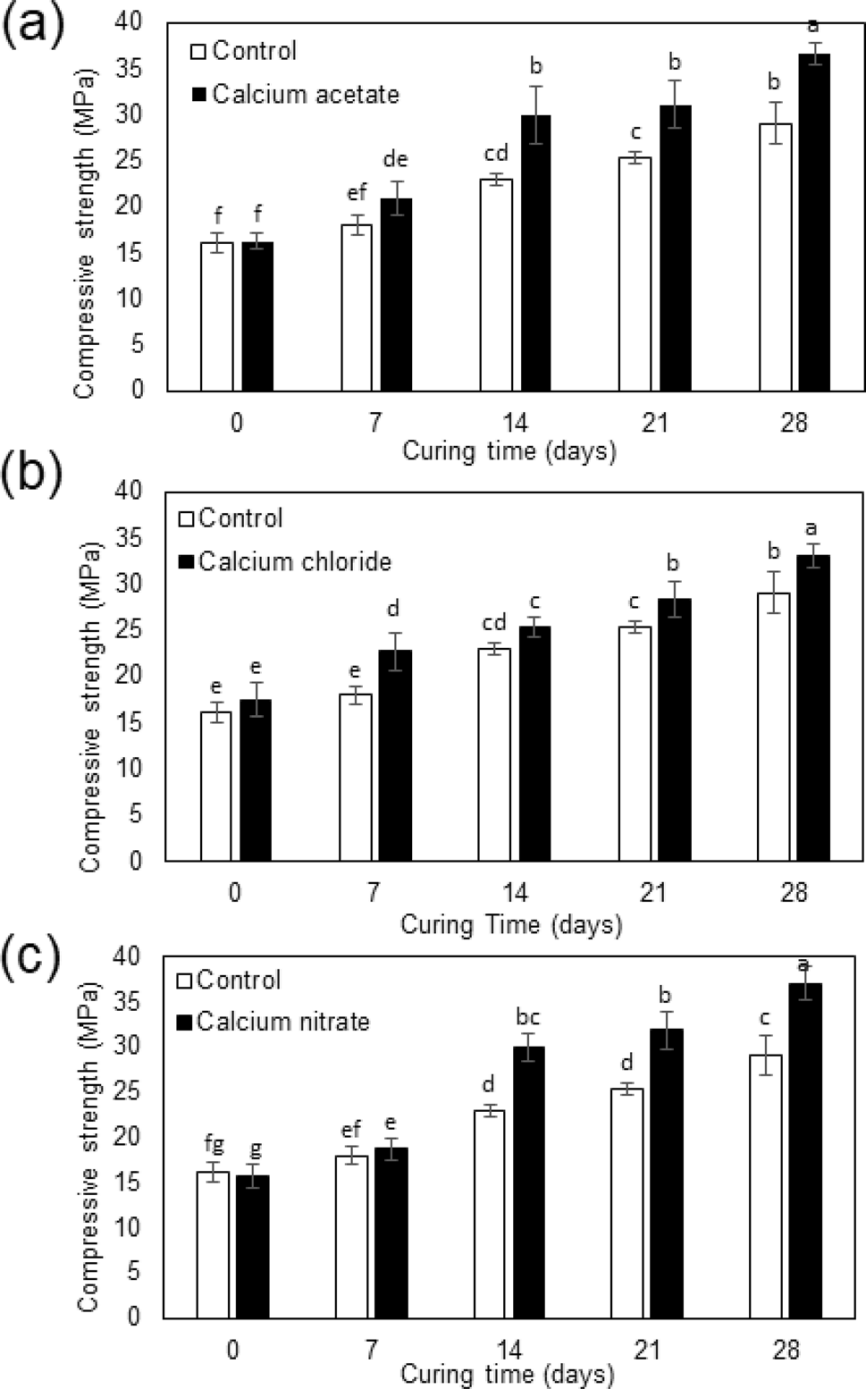
Compressive strength of biocement containing strain WH grown with different calcium sources—calcium acetate (**a**), calcium chloride (**b**) and calcium nitrate (**c**). Controls were non-inoculated cements. Error bars show standard deviations of data from triplicate experiments. Different labelling letters above the bars indicate significant difference at p-value < 0.05 when compared by using Least Significant Difference test (LSD). The data with the same letter are not significantly different.

The compressive strength of cement cubes containing strain WH grown with different calcium sources is presented in Fig. 7. According to statistical analysis, we found that after 14 days of curing time, the strength of biocement became significantly higher than that of the control in all conditions. On day 28, the compressive strength of biocement was 36.62, 31.86, and 37.02 MPa in cases of using calcium acetate, calcium chloride, and calcium nitrate as calcium sources, respectively. These values were significantly higher than that of the control which was only 29.05 MPa. In other words, strain WH could strengthen cement up to ~ 27.4% when using calcium nitrate as a calcium source. Likewise, a relatively high %increase in strength was found when using calcium acetate as a calcium source which was up to ~ 26.1%. In contrast, calcium chloride was not a suitable calcium source for strain WH to substantially strengthen cement (increased strength up to ~ 9.7%). Therefore, in terms of actual application, the use of calcium acetate or calcium nitrate for biocalcification by strain WH could better strengthen cementitious materials than the use of calcium chloride. The XRD and SEM results of cement were also provided in the Supplementary Figures 2 and 3.

## Discussion

Although strain WH could grow in the presence of all calcium sources tested in this work, the highest growth and pH was found in the case of calcium acetate (Fig. 3). The pH also increases with increasing the bacterial biomass, indicating that our strain could induce an alkaline environment supporting CaCO_3_ precipitation. Note that, in principle, Ca is not the main constituent for microbial growth but the calcium source is provided to be the source of Ca ions. The bacteria can form carbonate ions by using C and O from such as dextrose which are also available in standard culture medium. The results suggested that calcium acetate was the best calcium source to promote growth (up to ~ 7 × 10^10^ cfu/ml) and CaCO_3_ production. This is because calcium acetate can serve as both Ca and C source for bacterial growth, which then resulted in higher amounts of nucleation sites for CaCO_3_ formation. We found that CaCO_3_ production, which were 0.8, 0.2, and 0.6 g/l, in cases of calcium acetate, calcium chloride, and calcium nitrate, respectively, (Fig. 4) were comparable to those obtained from other related genera. For example, *B. licheniformis* AK01^33^ and *Bacillus* spp.^34^ produced ~ 0.9–1.0 g/l of CaCO_3_ when grown in calcium acetate and calcium chloride, respectively. Nevertheless, recent study^35^ showed that *Sporosarcina pasteurii* grown in a urea-containing medium could produce CaCO_3_ as high as ~ 6.7 g/l. Although the main focus of this work is on the elastic properties of our biogenic CaCO_3_ and its application to biocement, investigating the optimum condition to increase the amount of CaCO_3_ is planned for the future.

The use of different bacterial strains to precipitate CaCO_3_ for biocement application has been previously reported. Achal et al.^36^ showed that *Sporosarcina pasteurii* can be used for application of cement mortar and concrete. Some bacteria are also capable of remediation of cracks in concrete (e.g., *Bacillus pasteurii*^37^, *Bacillus pseudofirmus*^11^, and *Bacillus sphaericus*^15^). Among these bacteria, the most effective CaCO_3_ producer was *Bacillus sphaericus* but it showed relatively high urease activity^15,38^. Meanwhile, our strain WH could produce CaCO_3_ in the absence of urea, suggesting that a non-desired byproduct via urea hydrolysis that may affect steel corrosion in reinforced concrete can be avoided.

Since calcium source is one of the main factors altering bacterial cell surface properties, our strain could induce the formation of not only calcite but also vaterite (a metastable form of calcite) depending on types of calcium sources (Fig. 5). Previous studies, for example by Bentz et al.^39^, suggested that calcites could promote the cement hydration process, while aragonite (another form of CaCO_3_) could not. This is because both Ca and O atoms can be seen in the surface layer of calcite that match the CaO layer usually found in calcium silicate hydrate (or C–S–H), the dominant binding phase in Portland cement hydrates. In the case of aragonite, only Ca atoms are seen in its planar configuration. The results showed that strain WH grown with calcium chloride and calcium nitrate could produce calcite, suggesting its potential to be used for accelerating the hydration process that produces C–S–H which is the main source of cement strength. On the other hand, CaCO_3_ obtained from calcium acetate cultures, which is in the vaterite phase, might be less compatible with cement when compared to calcite from the other two sources. Due to being less stable, vaterite can possibly change to calcite structure or monohydrocalcite (CaCO_3_⋅H_2_O)^40^ during the cement preparation process. Nevertheless, monohydrocalcite, whose mechanical properties are weaker than calcite and vaterite, would be rarely observed because it is least stable among other forms^41^. Therefore, CaCO_3_ from calcium acetate cultures would be more suitable for biocementation, if it can be kept stable in the vaterite phase or completely transformed to calcite phase^42^.

Now we first compared the elastic parameters of our biogenic CaCO_3_ to those of the limestone cement. Limestone cement typically has a density of 2700–2800 kg/m^3^, the bulk modulus of elasticity of 69.8 GPa, the Young's modulus of 79.6 GPa, the shear modulus of 30.4 GPa and Poisson's ratio of 0.31^43,44^. According to Tables 2 and 3, the elastic moduli of CaCO_3_ obtained from strain WH when using calcium nitrate and calcium chloride as a calcium source is comparable to those of the limestone cement. This indicated that our CaCO_3_ sample in calcite phase can be used as a mixture of cement in the same quality of common limestone. While CaCO_3_ in hexagonal vaterite phase obtained from strain WH using calcium acetate as a calcium source has elastic moduli lower than that of calcite phase around 15–20%, the Poisson’s ratio is higher than the other phase around 5%. Therefore, biogenic CaCO_3_ in the vaterite phase shows more ductile behavior when compared to the calcite phase. Although two different structures of CaCO_3_ were obtained, their mechanical properties are still in the range of applicability when compared to the quality of the commercial CaCO_3_ from Korth Kristalle GmbH (Kiel, Germany)^45^.

In addition, we also compared the elastic parameters of biogenic CaCO_3_ to those of the Portland cement, which is the most common type of cement used as a mixture of concrete, mortar and stucco. Portland cement has a density of ~ 3150 kg/m^3^, which is higher than that of limestone cement. The elastic properties which are commonly found in Portland cement are: the bulk modulus of elasticity of 40.0 GPa, the Young's modulus of 42.3 GPa, the shear modulus of 16.0 GPa and the Poisson's ratio of 0.324^46,47^. These values are in agreement with those of the main important phases (alite, belite and portlandite) presented in Portland cement pastes which obtained using force field atomistic methods^48^, and are 40–50% lower than those of predicted properties of our CaCO_3_ in the calcite phase. In other words, our biogenic CaCO_3_ are more durable than typical Portland cement around 40–50%. Furthermore, the predicted Poisson's ratio is still in the range of ductility material behavior, suggesting its potential to strengthen Portland cement while maintaining its ability to deform under applied loading. In the case of CaCO_3_ from strain WH grown with calcium acetate, the mechanical properties were in good agreement with those of vaterites previously reported^49^. Although these values are lower than those in the calcite phase, they are still significantly higher than those of Portland cement.

In terms of thermal expansion, the coefficient of thermal expansion (CTE) of the cement paste is 20 μs/°C^50^ that could increase as self-desiccation proceeds. The CTE of CaCO_3_ crystal is usually around 24 μs/°C^45^ which is comparable to that of the cement paste. Incorporation of nano-calcium carbonate in cementitious materials could also improve the compressive stress, strain, and toughness under ambient environment or even after high temperature exposure^19,51^. Note that the SEM micrographs and XRD spectra of uninoculated cement and biocement are shown in Supplementary Figures 2 and 3, respectively. Although the growth environments in medium and cement are different, similar trends of which there are different crystal morphologies between biocement and uninoculated cement could still be observed. There are more needle stick ettringite crystals (AFt) scattered in biocement than in uninoculated cement. The AFt can fill in the gap of biocement and help increase the compactness of the cement^52^. Note that the peaks of biogenic CaCO_3_ crystals could not be seen clearly from the XRD spectra of cement because less than 5% by weight is added in the biocement samples which is the standard amount usually used in previous literature^53,54^. This, however, could be beneficial in terms of saving the cost of biocement production and minimizing the effects of bacteria themselves on the biocement.

Note that ordinary Portland cement can be composed of different phases. The four main crystalline phases are C3S, C2S, C3A and C4AF. Among these, C3S is the major phase that could have a very high Young modulus of ~ 100 GPa which commonly plays the main role in cement strength. In principle, CaCO_3_ in cement is produced naturally through the carbonatation process which requires Ca(OH)_2_ as a reactant. The Reference Intensity Ratio (RIR) analysis^30^ from XRD results of cements showed that amounts of C3S is quite similar in all uninoculated cement and biocement samples with the weight fraction of ~ 16–19% (see Supplementary Table 1). However, in cases of biocement, we found relatively small amounts of Ca(OH)_2_ compared to the cases of uninoculated cement. Contrarily, the amounts of CaCO_3_ are relatively high in biocement samples. Furthermore, the SEM results of cement showed different crystal morphologies in biocement and uninoculated cement. Therefore, an increase in CaCO_3_ in biocement samples could be induced by our bacterium. These CaCO_3_ could also serve as the nucleation matrix that could promote the carbonation and hydration process^51,52^. Additional CaCO_3_ produced by strain WH then should help enhance the mechanical property of cement-based composites.

Finally, the compressive strength of inoculated and non-inoculated cement cubes was investigated to prove the potential application of strain WH as a mixture in Portland cement. Although the largest amount of biogenic CaCO_3_ was found when using calcium acetate, the highest compressive strength of 37.02 MPa (with an increase of 27.4% compared to the non-inoculated cement) was observed in biocement using calcium nitrate. This should be because CaCO_3_ were in the form of calcite, which is more durable than typical Portland cement. However, strain WH grown with calcium chloride could increase the compressive strength of cement up to only 9.7%, despite the predicted elastic parameters being similar to those of calcium nitrate case. This is probably because strain WH could produce a significantly lower amount of CaCO_3_ when grown with calcium chloride (see Fig. 4). Such low quantity is not sufficient to improve mechanical properties of cement, otherwise the production of CaCO_3_ in this case needs to be upscaled, which would result in a higher cost of biocement production. Also in the real application, using calcium chloride can cause electrochemical corrosion of the steel reinforcement due to chloride penetration^3^. Therefore, calcium nitrate is the most appropriate calcium source for biocement production using strain WH.

Recently, an increase in cement strength of 17% was reported when using *Lysinibacillus sphaericus* grown with calcium acetate^54^. We remark that when using calcium acetate, strain WH could promote the compressive strength of Portland cement as high as 26.1%. In this case, the precipitated CaCO_3_ is in the form of vaterite, but its ability to strengthen cement is comparable to the case of calcite produced using calcium nitrate. This suggested that vaterite possibly changed to calcite structure during the cement preparation process carried out at low temperature, as also pointed out by e.g., Zhou et al.^31^. Further investigation of how our biogenic CaCO_3_ affects other physical properties of cement (e.g., water absorption by submersion and radiation protection) is planned for the future. To the best of our knowledge, there is no long-term study on the impact of bacteria themselves to the biocement. Keeping in mind relatively small amounts of bacterial biomass normally used in biocement experiments, the effects of bacteria themselves on the cement should be small at the beginning of experiments, but, however, is worth investigating in the future.

## Conclusion

A calcifying bacterium *Lysinibacillus* sp. strain WH was isolated from saline soil. Effects of different calcium sources including calcium acetate, calcium chloride and calcium nitrate, on the growth and CaCO_3_ precipitation were investigated. Although calcium acetate was the best calcium source to promote growth and CaCO_3_ production, calcium nitrate could also facilitate the production of a relatively high amount of CaCO_3_. In contrast, in the case of calcium chloride, poor growth and low level of CaCO_3_ production were obtained. The ANNs method was employed to analyze the crystal structures of CaCO_3_ from the experimental XRD spectra. The results showed that CaCO_3_ crystal from calcium acetate cultures was in a hexagonal phase of vaterite, while the cases of calcium chloride and calcium nitrate were calcite. Our analysis based on the VRH approximation suggested that the mechanical properties of biogenic CaCO_3_ are relatively similar to those of the limestone cement. Interestingly, they are significantly more durable than typical Portland cement around 40–50%. According to the biocement experiment, the highest increase in compressive strength (27.4%) was found in biocement containing strain WH grown using calcium nitrate as a calcium source. This improvement was comparable to other previous studies and even higher in some cases. Moreover, the compressive strength of Portland cement could also increase up to 26.1% when using calcium acetate as a calcium source. This might be because vaterite previously obtained changed to calcite structure during the cement preparation process. Both calcium nitrate and calcium acetate could be the calcium sources for strain WH to effectively strengthen Portland cement. Moreover, the ability of strain WH to produce CaCO_3_ via non-ureolytic processes favors their use as an alternative biomaterial for engineering since non-desired by-products occurred during urea hydrolysis can be avoided. Due to its advantageous characteristics, our newly-isolated *Lysinibacillus* sp. strain WH could produce CaCO_3_ for applying in Portland cement based materials.

## Supplementary information

Supplementary Information.
