# Supplementary material for: Investigating mechanical properties and biocement application of CaCO3 precipitated by a newly-isolated Lysinibacillus sp. WH using artificial neural networks
Source: Sci Rep. 2020 Sep 30;10:16137. doi: 10.1038/s41598-020-73217-7 (PMC7527966; doi:10.1038/s41598-020-73217-7)

**Manuscript title**: Investigating mechanical properties and biocement application of CaCO_3_ precipitated by a newly-isolated *Lysinibacillus* sp. WH using artificial neural networks

**Authors**: Jindarat Ekprasert^1,*^, Ittipon Fongkaew^2^, Poemwai Chainakun^2^, Rungtiwa Kamngam^1^, Wachiraya Boonsuan^1^

**Affiliations**: ^1^Department of Microbiology, Faculty of Science, Khon Kaen University, Thailand 40002

^2^School of Physics, Institute of Science, Suranaree University of Technology, Nakhon Ratchasima, Thailand 30000

**Supplementary Table 1**: Reference Intensity Ratio (RIR) analysis of crystal phases in cement samples

| Sample | Weight fractions of each phase in cement samples (%) | | |
| --- | --- | --- | --- |
|  | Portlandite (Ca(OH)_2_) | Calcite (CaCO_3_) | Hatrurite (C3S) |
| Uninoculated cement with calcium acetate medium | 60.1 | 22.5 | 17.3 |
| Uninoculated cement with calcium chloride medium | 57.3 | 22.9 | 19.8 |
| Uninoculated cement with calcium nitrate medium | 59.3 | 21.5 | 19.2 |
| Biocement with calcium acetate medium | 50.7 | 31.9 | 17.4 |
| Biocement with calcium chloride medium | 51.8 | 32.3 | 15.9 |
| Biocement with calcium nitrate medium | 54.4 | 29.4 | 16.2 |

**Supplementary Figure 1**: Scanning electron micrographs (5,000X magnification) of the freeze-dried biogenic CaCO_3_ crystals. Each panel corresponds to different calcium sources - (a) calcium acetate, (b) calcium chloride, and (c) calcium nitrate. Scale bars are shown at the bottom right.


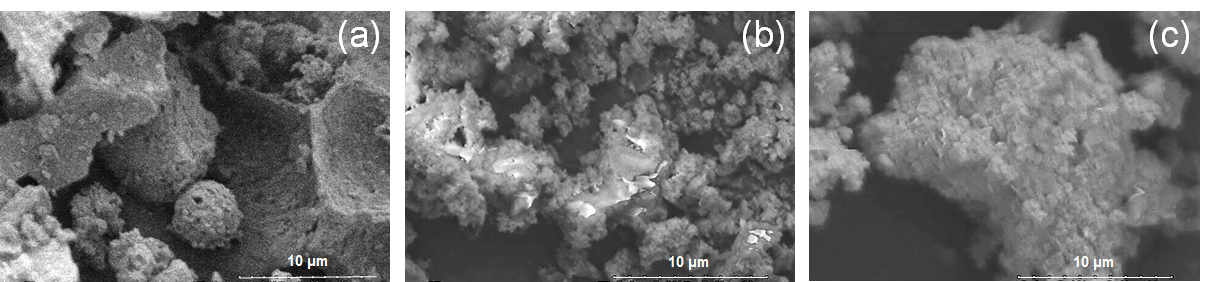


**Supplementary Figure 2**: Scanning electron micrographs (15,000X magnification) of biocement samples inoculated with *Lysinibacillus* sp. strain WH grown with calcium acetate (d), calcium chloride (e) and calcium nitrate (f), compared to uninoculated cements in their corresponding controls showing in (a), (b) and (c), respectively. Each picture is a representative of the multiple similar pictures. Scale bars are shown at the bottom right.


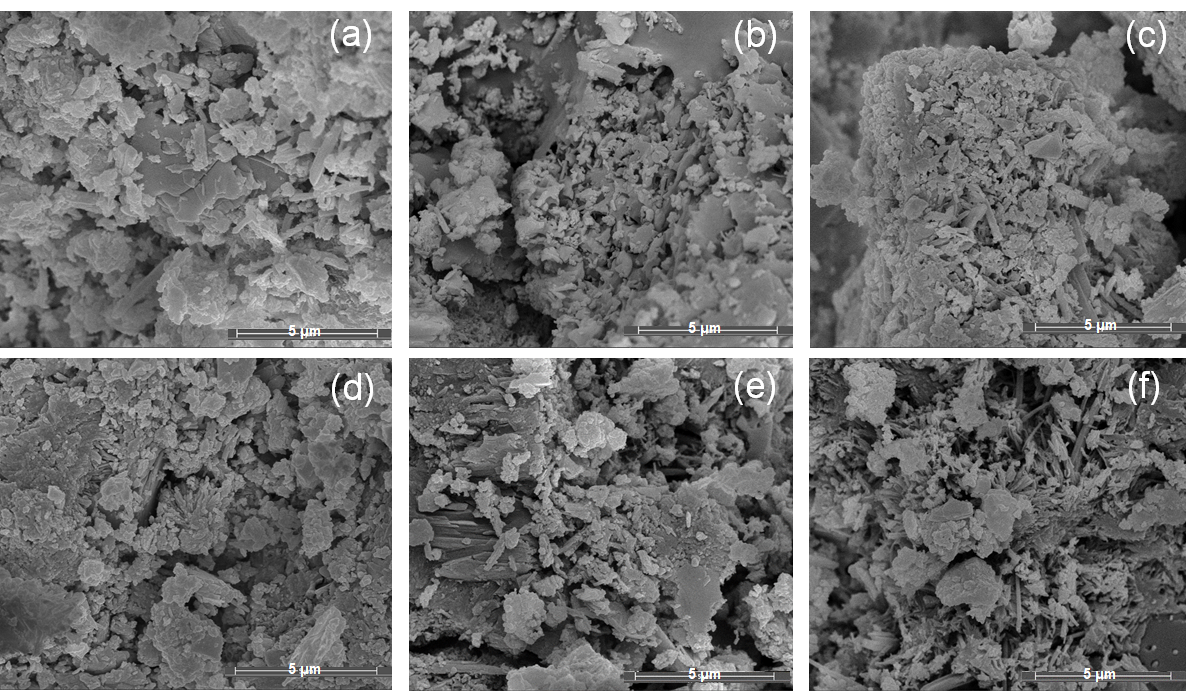


**Supplementary Figure 3**: XRD spectra of biocement samples inoculated with *Lysinibacillus* sp. strain WH grown with calcium acetate (d), calcium chloride (e) and calcium nitrate (f), compared to uninoculated cements in their corresponding controls showing in (a), (b) and (c), respectively.


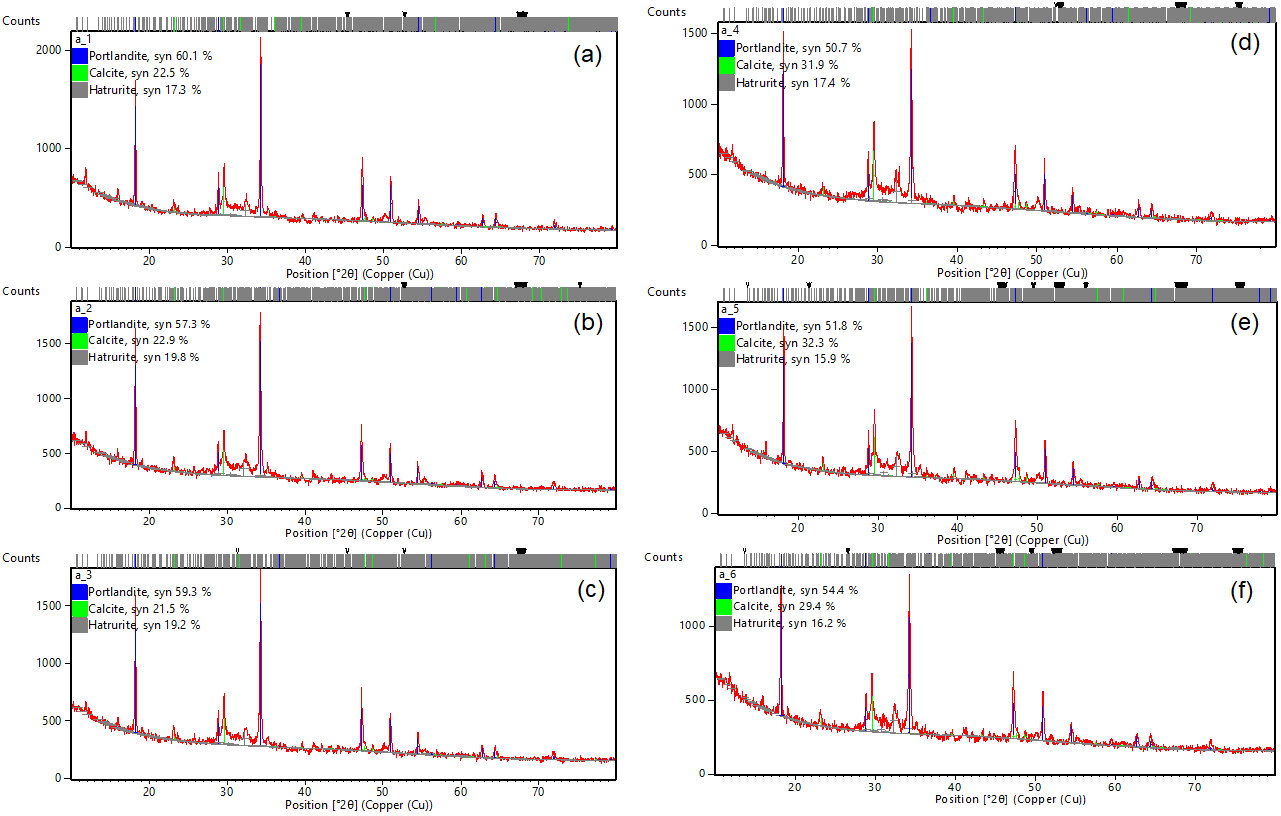

Supplement: Supplementary file 1 — Supplementary Information. [file 41598_2020_73217_MOESM1_ESM.docx]
